# Enantioselective Detection of Gaseous Odorants with Peptide–Graphene Sensors Operating in Humid Environments

**DOI:** 10.1021/acsami.4c01177

**Published:** 2024-04-03

**Authors:** Yui Yamazaki, Tatsuru Hitomi, Chishu Homma, Tharatorn Rungreungthanapol, Masayoshi Tanaka, Kou Yamada, Hiroshi Hamasaki, Yoshiaki Sugizaki, Atsunobu Isobayashi, Hideyuki Tomizawa, Mina Okochi, Yuhei Hayamizu

**Affiliations:** † Department of Materials Science and Engineering, School of Materials and Chemical Technology, Tokyo Institute of Technology, 2-12-1 Ookayama, Meguroku, Tokyo 152-8550, Japan; ‡ Department of Chemical Science and Engineering, School of Materials and Chemical Technology, Tokyo Institute of Technology, 2-12-1 Ookayama, Meguroku, Tokyo 152-8550, Japan; § Corporate Research & Development Center, Toshiba Corporation, 1, Komukai-Toshiba-Cho, Saiwai-ku, Kawasaki 212-8582, Japan

**Keywords:** peptide, bioelectronic nose, biogenic volatile organic compounds, graphene field effect transistor, enantioselectivity, biosensor

## Abstract

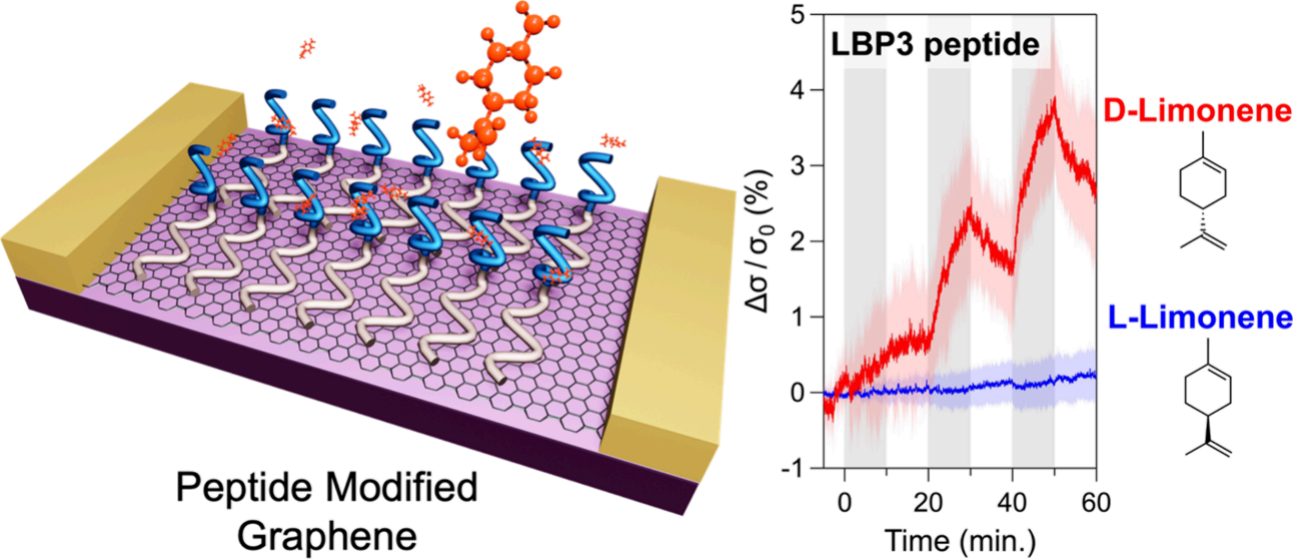

Replicating the sense of smell presents an ongoing challenge in the development of biomimetic devices. Olfactory receptors exhibit remarkable discriminatory abilities, including the enantioselective detection of individual odorant molecules. Graphene has emerged as a promising material for biomimetic electronic devices due to its unique electrical properties and exceptional sensitivity. However, the efficient detection of nonpolar odor molecules using transistor-based graphene sensors in a gas phase in environmental conditions remains challenging due to high sensitivity to water vapor. This limitation has impeded the practical development of gas-phase graphene odor sensors capable of selective detection, particularly in humid environments. In this study, we address this challenge by introducing peptide-functionalized graphene sensors that effectively mitigate undesired responses to changes in humidity. Additionally, we demonstrate the significant role of humidity in facilitating the selective detection of odorant molecules by the peptides. These peptides, designed to mimic a fruit fly olfactory receptor, spontaneously assemble into a monomolecular layer on graphene, enabling precise and specific odorant detection. The developed sensors exhibit notable enantioselectivity, achieving a remarkable 35-fold signal contrast between d- and l-limonene. Furthermore, these sensors display distinct responses to various other biogenic volatile organic compounds, demonstrating their versatility as robust tools for odor detection. By acting as both a bioprobe and an electrical signal amplifier, the peptide layer represents a novel and effective strategy to achieve selective odorant detection under normal atmospheric conditions using graphene sensors. This study offers valuable insights into the development of practical odor-sensing technologies with potential applications in diverse fields.

## Introduction

Biogenic volatile organic compounds (BVOCs) are a kind of volatile organic compound synthesized predominantly in plants. BVOCs have become more important in several fields, such as health care, environmental monitoring, food, and the agricultural industry.^1,2^ For example, the emission of BVOCs to air from plants is closely related to the environments and climates^3,4^ and is also associated with plant diseases.^5−7^ Thus, the detection of BVOCs in air is crucial for the above field to monitor the environmental conditions of plants. Furthermore, the detection of BVOCs at customs in airports and harbors is becoming more important for plant protection and quarantine. It is desirable to develop automated systems to detect plants and related materials on behalf of quarantine dogs.^8,9^

The sensing of the BVOCs has been conducted by gas chromatography–mass spectrometry (GC-MS), quartz crystal microbalance (QCM), and surface plasmon resonance (SPR) in the past. Although the GC-MS is the most reliable and common technique,^10,11^ the equipment is relatively large, and small and mobile devices are preferable for ubiquitous usage at farm fields or border customs. QCM^12^ and LSPR^13^ sensors can be relatively small in their dimensions. The surface functionalization of these sensors is the key to achieving the selective detection of BVOCs. The molecular imprinting technique and ionic liquid immobilization exhibited significant progress in the selective detection of BVOCs, and there are several demonstrations of monitoring BVOCs in the air.^14−16^ However, considering applications in quality control of plants in farms and quarantine at airports, it is still challenging to develop a sensing system that satisfies (1) small size, (2) stability against ambient conditions containing water vapor, (3) low power consumption, and (4) high sensitivity and selectivity.

Graphene has attracted much attention as an active material for sensing due to its high mobility, large specific area, and accessibility for surface modification. The successful detection of 1 ppm NO_2_ and water molecules was achieved using graphene field effect transistors (GFETs).^17^ To date, GFETs have demonstrated the detection of multiple volatile organic compounds, such as NH_3_,^18^ CO_2_,^18,19^ and NO_2_.^20,21^ More recently, GFET-base sensors identify “odor molecules” combined with machine learning to selectively obtain molecule-specific responses.^22,23^ Selective sensing has also been improved by modifying the graphene surface with polymers^24^ and ssDNA.^25^

Despite this progress, GFET-based BVOC sensors require further improvement in more practical environments where stable operation under various humidity levels is required. So far, graphene has been reported to respond to water vapors at high sensitivity (1 ppm level).^17^ The electrical and chemical properties of pristine graphene depend on the humidity in the environment.^26,27^ Furthermore, transistor-based graphene sensors have difficulty detecting nonpolar molecules, i.e., most odorant molecules. Therefore, it is crucial to establish a novel interface between graphene and atmospheric air to achieve a stable operation while simultaneously maintaining high selectivity and sensitivity simultaneously.

In this study, we present a novel method for detecting BVOCs under humid conditions using peptides designed to functionalize the surface of graphene sensors. Our peptides consist of an assembly domain and bioprobe for the detection of BVOCs. Specifically, our peptide design includes probe domains that mimic the olfactory receptor of fruit flies, which have high selectivity for limonene. The probe domain connects with an assembly domain that can form ordered structures on graphene surfaces in a self-assembly manner, allowing for the formation of a uniform peptide layer in a simple way. Unlike untreated graphene sensors, our peptide-functionalized graphene sensors showed a stable response to the BVOCs even under humid conditions. We achieved 35-fold contrast in the electrical signal for enantioselective detection of limonene. Interestingly, the sensors selectively detected enantiomers only in the presence of water vapor. We also tested three different peptide probes and obtained distinct electrical responses for multiple kinds of BVOCs. Principal Component Analysis (PCA) and Hierarchical Cluster Analysis (HCA) confirmed this trend. Our results demonstrate the potential of peptide-engineered graphene sensors as a versatile tool for selective BVOC detection under humid conditions.

## Results and Discussion

### Functionalization of Graphene Surfaces by Peptides

Developing the bioprobe is the key to producing more practical odor sensors. The range of the probes spans from synthetic molecules and polymers^28^ to engineered proteins such as olfactory receptors.^9^ The flat surface and high surface area of graphene are suitable for surface functionalization and characterization. Owing to these advantages, the high sensitivity and selectivity of graphene odor biosensors have been achieved by integrating the bioprobes immobilized on the graphene surface.^29−31^ Olfactory receptors as a bioprobe have demonstrated outstanding sensitivity and selectivity against amyl butyrate.^32,33^ However, the fragility of these proteins hinders the long-term stability of the graphene odor sensors. Peptides are an alternative candidate that mimics the olfactory receptors’ properties and have demonstrated odor sensing using multiple platforms.^34^

Our recent work has demonstrated the selective detection of odor molecules using GFETs functionalized with insect olfactory receptor mimicking peptides, where the graphene odor sensors were operated under liquid conditions.^35^ In this work, we propose a strategy to operate GFETs for BVOC sensing in the gas phase. The challenge here is the stable operation of graphene odor sensors functionalized with peptides with high selectivity in a gas phase in the presence of water vapor. Graphene is generally sensitive to adsorbed polar molecules, including water molecules.^17,18,36^ It has been a significant obstacle to the use of graphene odor sensors in ordinary environments containing water vapor.

**Figure 1:**
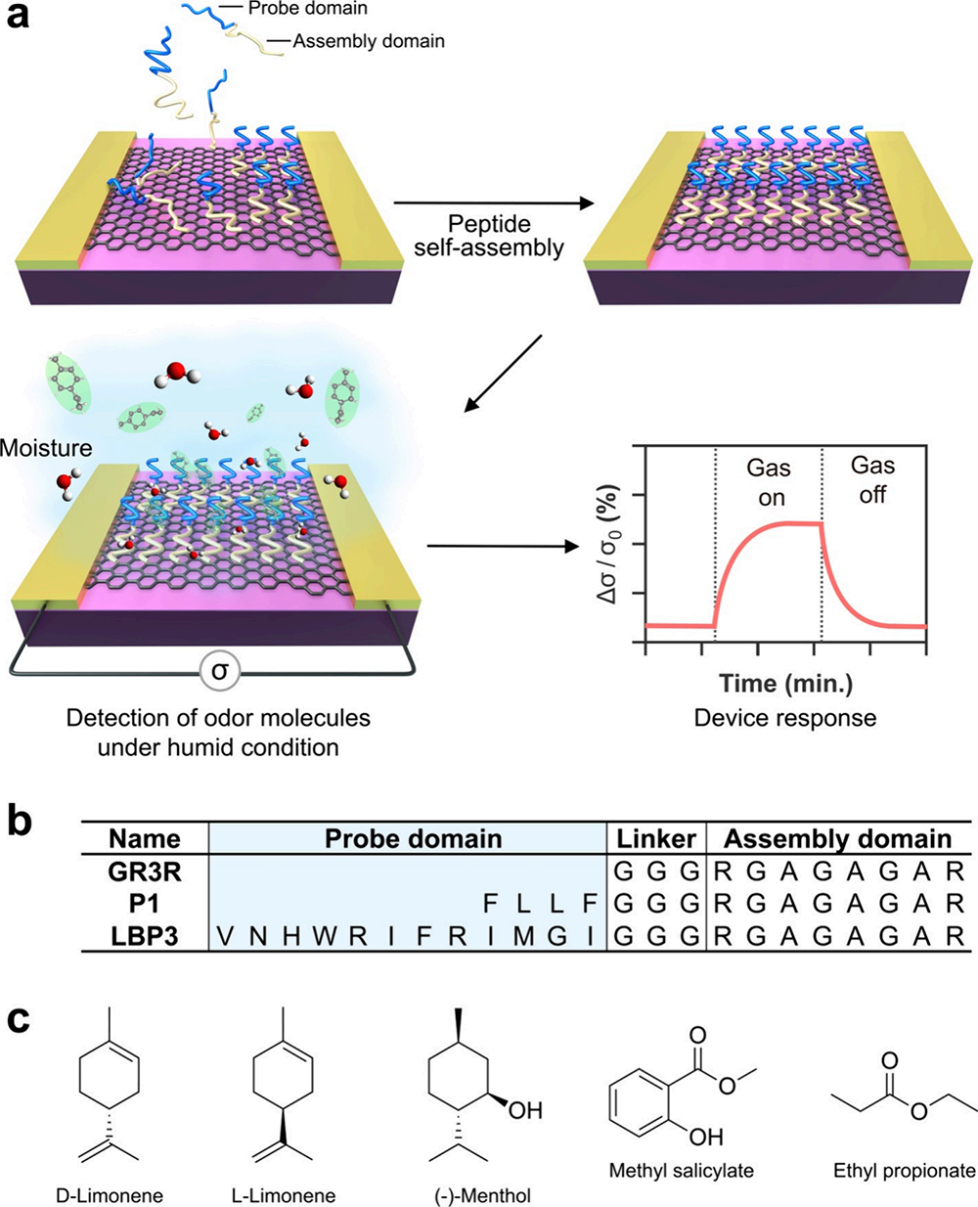
Surface functionalization of graphene biosensor for the detection of biogenic volatile organic compounds. (a) Schematic showing the peptide self-assembly on graphene and the peptide-graphene odor sensors operating in the presence of water vapor. (b) Peptide sequences. Peptides consist of three domains: probe, linker, and assembly domain. (c) Molecular structures of the biogenic volatile organic molecules used in this work.

Similar to prior studies,^35,37−39^ we employed peptides with self-assembly capability into molecular films on surfaces, accomplished through a series of surface processes involving adsorption, diffusion, and intermolecular interactions. Noncovalent interactions between the peptides and the surface facilitate their surface diffusion, eventually leading to their ordering along specific directions (Figure 1a). Peptides used in this work are named GR3R, P1, and LBP3 (Figure 1b). Our peptides are composed of two functional domains: (1) an assembly domain and (2) a probe domain. The assembly domain includes dipeptide repeats of glycine (G) and alanine (A), with two arginines (R) at both ends of the sequence. All peptides have GGG as a spacer for the bioprobes, in addition to the GA repeats. P1 and LBP3 share the assembly domain with the sequence of GR3R.^35,37^ P1 and LBP3 contain the probe domain as a bioprobe for the target odor molecules in addition to the assembly domain. Our recent findings^35^ highlighted that a mixed solution of P1 peptides and GR3R at several mixing ratios formed a coassembled peptide layer on a graphite surface with monomolecular thickness. Furthermore, the ordered molecular films were found to maintain their self-assembled structures stably even under liquid conditions. These bifunctional peptides are designed to create a uniform monomolecular thick layer on graphene, while the probe domain actively captures BVOCs on the surface. The resultant binding events can be electrically detected by a change in graphene conductivity.

The sequences of the probe domain are designed as follows. P1 peptide containing FLLF (F: Phenylalanine and L: Leucine) connected to the N-terminus of GR3R was rationally designed. This sequence mimics a part of the sequence of the olfactory receptor Or19a.^40−42^ The sequence of the LBP3 peptide was experimentally found as a strong binder of d-limonene.^38^ Short peptide libraries derived from OR19a were synthesized on a peptide array, and the amount of bound d-limonene on each peptide was analyzed, allowing a simultaneous exploration of the whole receptor protein.^38^ In the demonstration, we employed d-limonene, l-limonene, (-)-menthol, methyl salicylate, and ethyl propionate as target odor molecules to evaluate the selectivity of the proposed GFET gas sensor (Figure 1c).

First, we examined whether these peptides could form a thin film on the graphite surface through self-assembly. Atomic force microscopy (AFM) showed that GR3R peptide formed a uniform thin film on the surface with a thickness of about 1.5 nm (Figure S1), consistent with a previous work.^37^ P1 and LBP3 also exhibited the formation of thin films on the graphite surfaces.

### Effect of the Peptide Modification against Humidity

**Figure 2:**
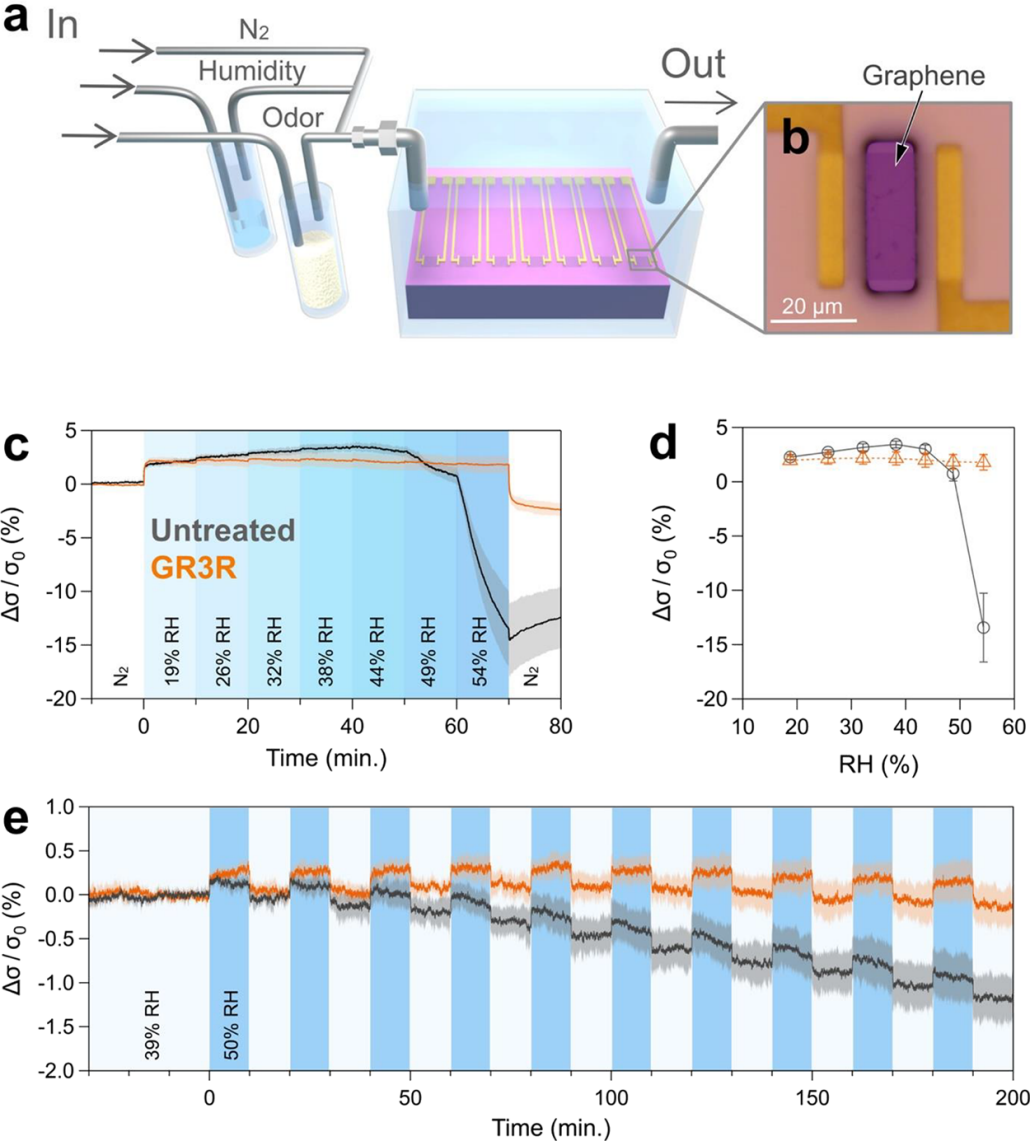
Graphene sensors responding to relative humidity. (a) Schematic of the gas measurement system for the graphene odor sensor with controlled humidity and flow rate of odor molecules. (b) Optical microscopic image of a graphene sensor chip. (c) Real-time response of graphene sensors to humidity change: untreated (black) and GFET functionalized with GR3R peptides (orange). The curves and light-colored regions represent the mean and standard deviation of the data points, respectively. (d) Conductivity change of graphene depending on the relative humidity derived from panel (c). The bars indicate the standard deviations. (e) Accelerated test of untreated (black) and GFETs functionalized with GR3R peptides (orange) responding to the repeated humidity change. The curves and light-colored regions represent the mean and standard deviation, respectively.

We evaluated the response of our peptide-modified odor sensor against the humidity. Three sensor chips were placed in series (Figure 2a). Three mass flow controllers regulate the flow rates of N_2_ carrier gases introduced to water cylinders and target odor molecules to produce each vapor independently. The mixture of gases was introduced into graphene odor sensors with controlled humidity and flow rates. An in-line humidity sensor monitored the relative humidity at the end of the gas line system (Figure S2). Each chip contains seven graphene channels with an open window of 10 μm× 30 μm between the source and drain electrodes (Figure 2b). The rest of the area was covered by a polyimide protection layer. The GFET chip was placed on a specified mount and encased within a well made of polytetrafluoroethylene (PTFE). This well was uniquely designed with two openings, serving as an inlet and an outlet for gases, thereby establishing a pathway for the gas flow to the graphene channel (Figure 2a).

Our graphene sensors had a field-effect transistor structure showing a reproducible gate response before and after the peptide functionalization (Figure S3). We studied the conductivity change of the GFET against humidity. Before introducing water vapor, the GFETs were purged with pure N_2_ gas for 80 min to initialize the surface state. The relative humidity (RH) was then increased incrementally from 19% to 54% RH every 10 min (Figure 2c). After introducing the first vapor gas at 19% RH, the conductivity of the untreated GFET increased by 1.6%. Conductivity increased monotonically with increasing up to 44% RH.

Further increases in humidity caused the GFET response to water vapor to become unstable, decreasing by −15% at 54% RH. The negative conductivity change remained even after the graphene surface was rinsed with N_2_ gas. In contrast, the GFET functionalized with GR3R showed a stable response to water vapor. After introducing carrier gas at 19% RH, the conductivity increased by 2% and remained constant even when the RH was increased to 54% (Figure 2d). Comfortable humidity levels for humans in public buildings are known to be between 40% and 60% RH. For the use of graphene odor sensors in such environments, a key issue with untreated GFETs is that their humidity response becomes unstable at around 50% RH. Peptide-functionalized GFETs, on the other hand, have proven their stability.

Accelerated tests of GFETs were also conducted against repeated changes in humidity at a constant level. Figure 2e shows the conductivity change under the repeated change of the humidity with 39% and 50% RH 10 times. Both untreated and peptide-functionalized GFETs exhibited a step-function-like response against the humidity change. Notably, the untreated GFET decreased the baseline of the conductivity by 3% after a 30-time repeat. On the other hand, peptide-functionalized GFET had a stable baseline. These results indicate that peptides act as stabilizers on the graphene surface against humidity change.

The conductivity change in graphene when exposed to water vapor has been extensively studied.^26,27,43−46^ The mechanism behind this change in conductivity due to adsorbed water molecules is multifaceted, potentially involving hole doping,^45^ band gap tuning,^43^ interaction with the SiO_2_ surface of the substrate,^44^ and intercalation of water molecules between the graphene and the substrate.^46^ The observed increase in conductivity (Figure 2c) is likely attributable to hole doping of graphene by water molecules. The significant decrease in conductivity at high humidity levels observed in untreated graphene sensors may result from a combination of these mechanisms. In contrast, sensors using peptide-functionalized graphene showed a remarkable insensitivity to changes in humidity. This is possibly because the peptides act as a buffer for water molecules, wherein the water molecules are absorbed into the peptide monolayer, maintaining stable electrical polarization within the dielectric medium of the peptides, even under varying humidity levels. This hypothesis is further corroborated by environmental AFM measurements of the self-assembled peptides. These measurements, conducted in a controlled humidity chamber, reveal an increase in peptide thickness under varying humidity conditions, as illustrated in Figure S4.

### Enantioselective Detection of Limonene

**Figure 3:**
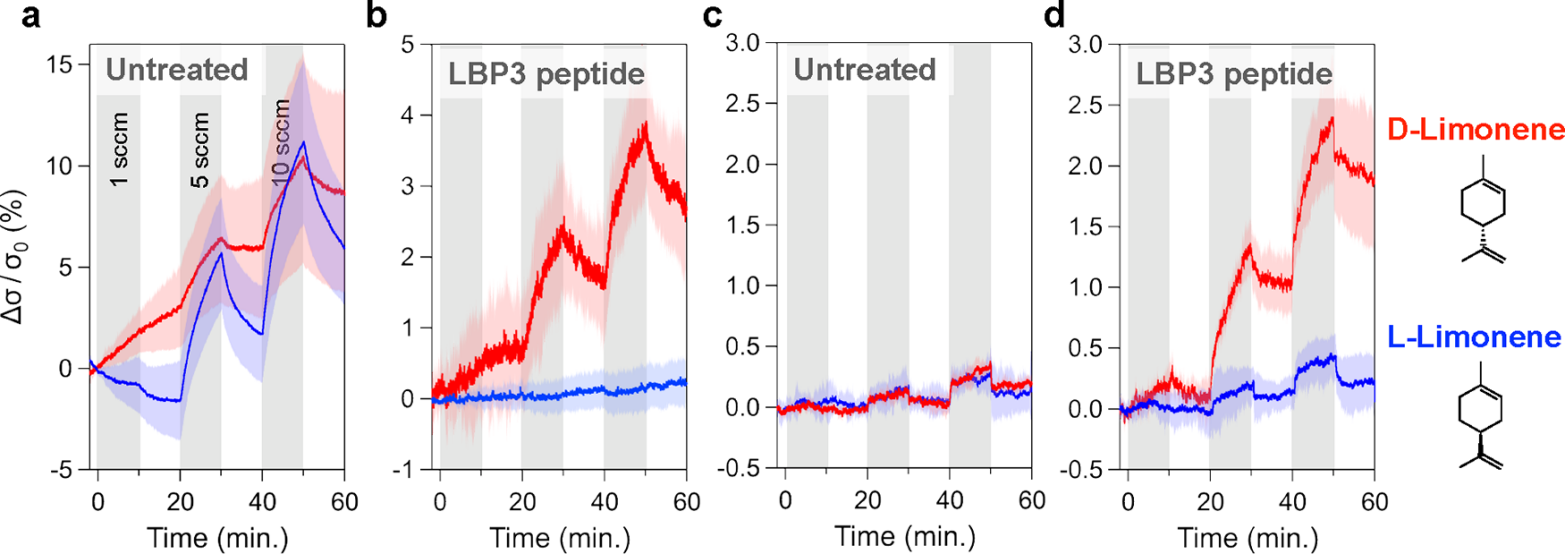
Chiral recognition by GFETs functionalized with peptides. Conductivity changes of GFETs responding to enantiomers of limonene under (a, b) 53% RH and (c, d) N_2_ conditions. The curves show the responses of GFETs to d-limonene (red) and l-limonene (blue). The chiral selectivity differed between untreated GFETs and those functionalized by LBP3 peptides. The curves and colored shadows represent the mean value and standard deviation, respectively.

Limonene is a molecule with two enantiomers that humans can selectively recognize. d-Limonene is perceived as a lemon scent, while l-limonene is perceived as a turpentine scent. Our peptide-functionalized GFETs were tested for detecting both d- and l-limonene (Figure 3). Prior to measurement, the GFET was exposed to a N_2_ carrier gas or 53% RH gas until the conductivity of the GFET became stable. Once a steady state was reached, limonene gas was injected three times at different concentrations of target molecules. The target gas injection lasted 10 min as the “ON” state, followed by 10 min of purging with 53% RH carrier gas as the “OFF” state. The odorant molecule gas flow rate was increased by 1, 5, and 10 sccm during each ON cycle. In this way, we evaluated the binding and desorption of limonene molecules.

The changes in the conductivity clearly illustrate the difference between the untreated and peptide-functionalized GFETs. In the untreated GFETs, the conductivity increased upon introduction of the enantiomers during the ON state. Subsequently, the conductivity gradually decreased upon switching to the OFF state. The average magnitudes of the conductivity changes were similar for d- and l-limonene (Figure 3a). On the other hand, peptide-functionalized GFETs responded only to d-limonene, with almost no signals for l-limonene. The difference in the conductivity changes between d- and l-limonene was 35-fold, demonstrating the significant enantioselectivity of LBP3 for d-limonene (Figure 3b).

The response of GFETs to d- or l-limonene vapors is intricately linked to the presence of water molecules. When tests were conducted under dry conditions, using only N_2_ gas, untreated GFET revealed the same responses to d- and l-limonene vapors (Figure 3c). Furthermore, LPB3 functionalized GFET had a notable decrease in selectivity was observed (Figure 3d and Figure S5). This outcome suggests that the molecular interactions between the peptides and odorant molecules are significantly influenced by the water molecule content at the graphene interface. The presence of water molecules likely imparts flexibility to the peptides, enabling them to capture odorant molecules in an energetically favorable manner, thereby enhancing the selectivity. This interface is optimally suited for use in normal air conditions, where humidity is present.

Previously, the enantioselective detection of odorant molecules using graphene sensors has been demonstrated with limited selectivity of less than 2-fold.^47^ The significant contrast observed in this work was achieved with the peptide probe. The fundamental role of the peptide layer in our GFETs is to differentiate between very similar molecules such as d-limonene and l-limonene in this case. The peptide-functionalized GFETs work on a mechanism in which the peptides specifically interact with the odorant molecules, causing a change in the electrical properties of the sensor. This interaction is highly dependent on the structural fit between the peptide and the odorant molecule. In our case, LBP3 peptides have a preferential binding toward d-limonene due to its specific structure (Figure S6), resulting in a more significant response for d-limonene than for l-limonene. This leads to a much higher selectivity; despite a reduced overall signal intensity, this effect is more pronounced under the conditions with humidity.

### Selective Detection for Other Odorant Molecules

**Figure 4:**
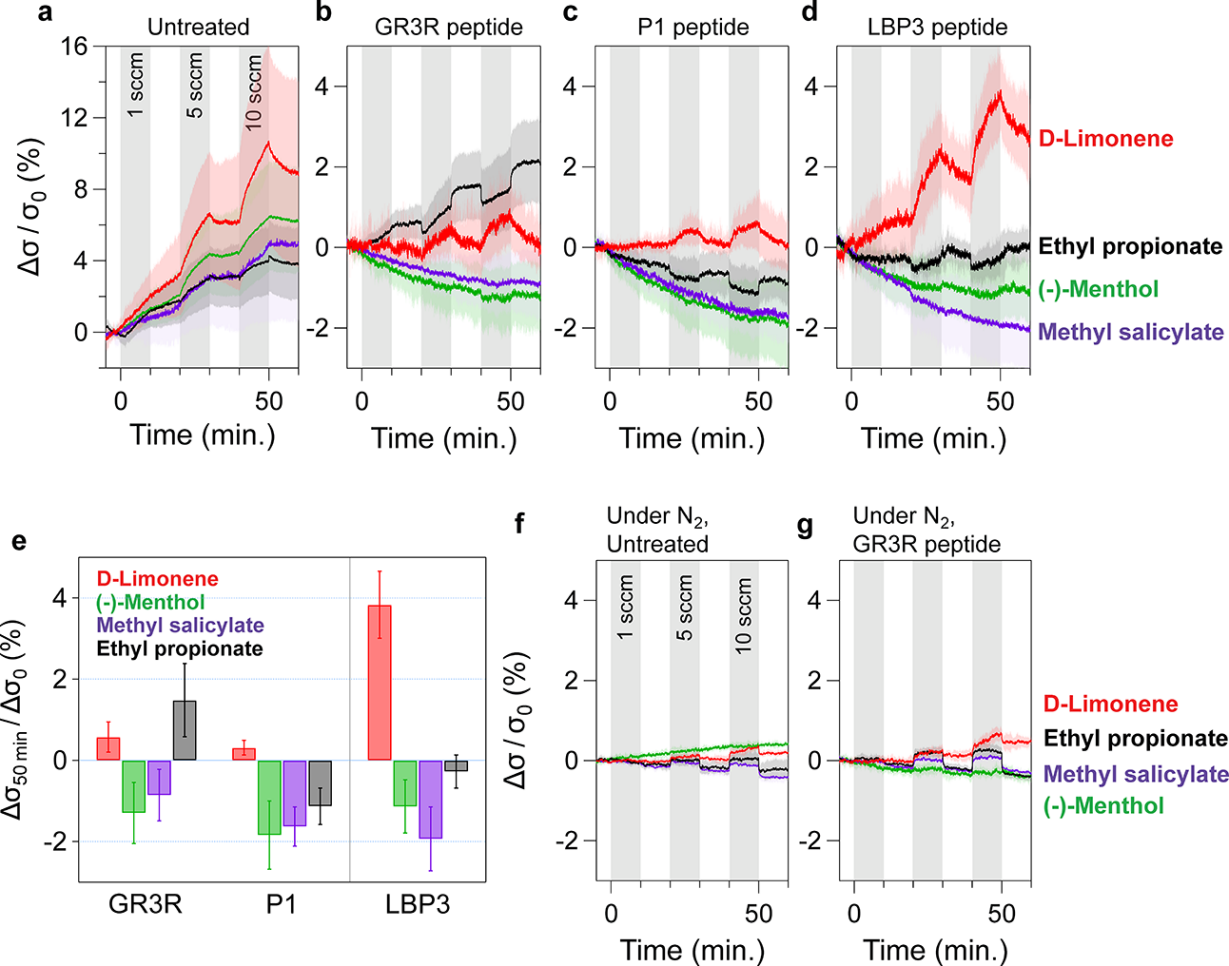
Real-time measurement of peptide-functionalized GFETs for each odorant molecule. Real-time response of GFETs to each odorant gas with incrementally increasing flow rates. Each plot shows the results of (a) untreated GFETs, and GFETs functionalized with (b) GR3R, (c) P1, and (d) LBP3. The colors of the curves represent the individual odorant gas: d-limonene (red), (-)-menthol (green), methyl salicylate (purple), and ethyl propionate (black). (e) Bar plot of the conductivity magnitudes at 10 min after 10 sccm of odorant gas injection. Real-time response of (f) untreated GFETs and (g) GFETs functionalized with GR3R peptides under N_2_ conditions. All curves represent the mean value of the conductivity change among the multiple channels.

We selected other odorant molecules to test the selectivity of the sensors. (-)-Menthol, methyl salicylate, and ethyl propionate are produced by plants in nature. Although these molecules have similar molecular weights, they smell distinctly like peppermint, root beer, and pineapple, respectively. In the electrical measurements, the ON and OFF states of the target molecules were examined at three different flow rates in the same manner as in Figure 3. Untreated GFETs exhibited relatively large responses in the conductivity for all odorant molecules, and there was no significant difference in their magnitudes among them (Figure 4a).

In contrast, peptide-functionalized GFETs showed a variety of responses for different odorant molecules (Figure 4b–d). Interestingly, (-)-menthol and methyl salicylate resulted in a decrease in conductivity, while d-limonene exhibited an increase in conductivity. Ethyl propionate had a more complex trend in the response, where GR3R increased conductivity but P1 and LBP3 decreased. In the case of GR3R, in particular, the change in conductivity during the ON state was negative, while the overall conductivity increased on average. Figure 4e summarizes the magnitudes of the responses in the GFETs against each target molecule, and Table S1 (Supporting Information) lists the vapor concentration at a flow rate of 10 sccm. First, LBP3 shows a significant selectivity. d-Limonene leads to a positive and large change in the conductivity, and other molecules have negative changes. In addition, GR3R and P1 have a small response to D-limonene.

The sign of the conductivity change, positive or negative, should have a strong correlation with the target molecule. As shown in a previous report investigating graphene sensors for the detection of various analytes,^22^ aromatic molecules tended to increase the conductivity of the graphene by their direct adsorption on the surfaces. This increase in the conductivity is consistent with our observations in the untreated GFETs, which show an increase in conductivity after introducing odorant molecules under pure N_2_ (Figure 4f) and 50% RH (Figure 4a) conditions.

The magnitudes of the responses in untreated GFETs were larger under humid conditions (Figure 4a) compared with dry conditions (Figure 4f), suggesting that the dipoles of water molecules at the graphene interface could enhance the signal along with the adsorption of odorant molecules. On the other hand, in the case of peptide-functionalized GFETs, the water effect is more crucial. The sign of the conductivity change was negative or positive depending on the odorant molecules. This phenomenon can be related to the peptide film on the surface, which can act as a receptor for the target molecules and a reservoir for water molecules. The absorbed odorant molecules can sensitively change the dielectric constant of the mixture of peptides and water molecules. The peptide film containing water molecules can be considered as a dynamic medium that responds to target molecules. Indeed, the responses of the untreated and peptide-functionalized GFETs were similar under dry conditions (Figure 4f,g). Notably, under humid conditions, the estimated limits of detection (LOD) of the untreated and LBP3 GFETs for d-limonene were 3.0 and 7.5 ppm, respectively. These LODs were smaller when measured under humid conditions than under dry conditions (Figure S7 and Table S2).

In this Discussion, we explore potential explanations for the distinctive features of peptide-functionalized GFETs. The interactions between graphene sensors and odorant molecules, along with the observed differences between untreated and peptide-functionalized GFETs, are primarily understood through the physicochemical properties of these molecules and specific sensing mechanisms. In untreated GFETs, the detection of various odorant molecules largely depends on their physical adsorption on the graphene surface. This adsorption process changes the charge distribution on graphene, consequently altering its conductivity. The magnitude of this change in conductivity is significantly affected by factors such as the hydrophobicity of the odorant molecule, its molecular size, polarizability, and affinity for the graphene surface.

In contrast, peptide-functionalized GFETs involve more specific interactions between the odorant molecules and the peptides. These peptides are chosen for their ability to selectively interact with certain odorant molecules. When an odorant molecule binds to a peptide, it triggers a conformational change in the peptide, impacting the local electronic environment of the graphene and thus changing its conductivity.

The difference in signal responses between untreated and peptide-functionalized GFETs for each specific odorant can be attributed to the enhanced selectivity provided by the peptides. These peptides not only selectively interact with specific odorant molecules but also reduce responses to other molecules. Based on these considerations, we interpret that this selectivity leads to more distinct signal responses to various odorants in peptide-functionalized GFETs compared to their untreated counterparts.

### Classification of Odorant Molecules by PCA

**Figure 5:**
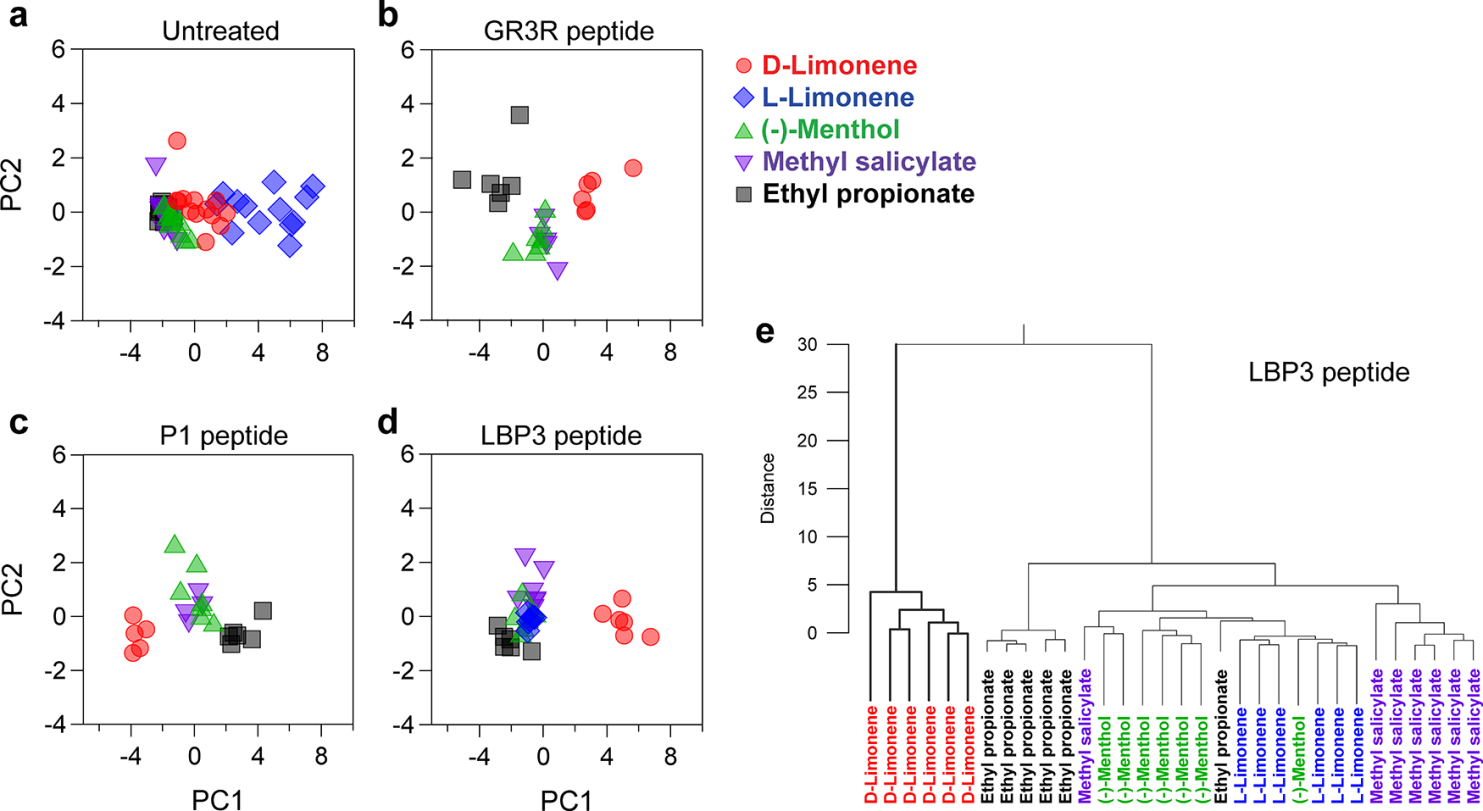
Discriminative detection of BVOCs by peptide-functionalized GFETs. (a–d) Principal component analysis score plots of GFETs for different odorants. (e) Dendrogram of LBP3 peptide generated by hierarchical cluster analysis.

To further analyze the selectivity of peptide-functionalized GFETs, we performed principal component analysis (PCA) and hierarchical cluster analysis (HCA) (Figures S8 and S9). PCA is an analytical method for building linear multivariate models from multidimensional data sets.^22,23,48^Figure 5 shows the results of the first two principal components. These contain > 86% of original data in their contributions (Figure S9). PCA results show a significant amount of overlap between d-limonene and other odorants in untreated GFETs, although l-limonene is relatively separated from the others (Figure 5a). Peptide-functionalized GFETs revealed considerable selectivity for some odorant molecules (Figure 5b–d). The LBP3 peptide separated d-limonene well from others along the PC1 axis. The GR3R and P1 peptides also separated ethyl propionate from (-)-menthol and methyl salicylate, indicating that these two peptides respond specifically to ethyl propionate. The PCA indicated that the LBP3 peptide was the most specific receptor to d-limonene.

In order to evaluate the selectivity more comprehensively, we also performed an HCA. HCA is a method that groups similar sample data into the same cluster in the space on the feature values.^48,49^ The distance between the samples measured the similarity of each sample. The dendrogram of the HCA results for peptide-functionalized GFETs shows the formation of an independent cluster of d-limonene. In contrast, the untreated GFETs do not discriminate specific BVOC (Figure 5e and Figure S10). This result is consistent with the PCA (Figure 5 a–d).

## Conclusions

This study introduces peptide-functionalized graphene sensors as a new approach for the selective and stable detection of odor molecules in the gas phase with humidity including the enantioselective recognition of limonene. Our results demonstrate that these sensors provide a stable response under different humidity conditions. LBP3, among the peptides tested, exhibited the highest magnitude of conductivity change in response to d-limonene, indicating its specific response as an artificial odorant receptor. The achieved enantioselective detection of limonene with a 35-fold contrast in the electrical signal is significant since previous studies on enantioselective detection of odorant molecules using graphene sensors have shown limited selectivity of less than 2-fold. Furthermore, our findings suggest that the presence of water molecules is the key to achieving remarkable selectivity. The LBP3 peptide can compete with odorant receptors in terms of enantioselectivity, which is promising for the development of biomimetic systems that have great potential for the understanding function of proteins^50^ and practical applications, such as plant monitoring in farms, quarantine in airports, and environmental monitoring. The diversity of peptide design also offers the opportunity to produce an array of sensors with multiple types of peptides, thus allowing for the tuning of analyte selectivity.^51^ Our study highlights the potential for biomimetic peptide functionalization of graphene sensors as a new approach for odorant sensing under normal air conditions with humidity.

## Experimental Section

### Peptide Preparation

GR3R and P1 peptides used in this study were purchased from the Toray Research Center, Inc. LBP3 peptide was synthesized with a purity higher than 90%. Peptide solutions were stored in a −20 °C freezer. Frozen peptide solutions were heated up to 70 °C for 15 min and allowed to reach room temperature.

### Atomic Force Microscopy (AFM) Measurements and Sample Preparation

In sample preparation for AFM measurements, Si substrates were first annealed on a hot plate at 200 °C for 30 min. After annealing, graphite flakes were transferred to Si substrates using a mechanical exfoliation method. 500-nM peptide solution was incubated for 1 h at room temperature. After incubation, the solution was removed by blowing with N_2_ gas.

The surface morphology of the peptide was characterized by using an atomic force microscope (Agilent 5500 and Asylum Cypher) in air. The surface morphology was measured in tapping mode (AC mode). The AFM instrument was equipped with a silicon cantilever (OMCL-AC160TS-R3, Olympus, Japan) with a resonance frequency of 300 kHz and a spring constant of 26 N/m.

### GFET Fabrication

Graphene was synthesized by chemical vapor deposition (CVD) and transferred to a Si wafer. Graphene and electrodes (50 nm Au and 5 nm Ti) were patterned by photolithography. Whole GFET chips were covered with polyimide as a protective shield, and only the graphene channels were exposed in the open window. The distance between the electrodes was 20 μm, and the width of the graphene was 30 μm. Each chip has seven channels, and we measured all channels simultaneously.

### Peptide Functionalization of the GFET Chip

500-nM peptide solution was placed on the graphene surfaces of the GFET chip at room temperature and kept for one hour. After incubation, the solution was removed by blowing with N_2_ gas.

### Materials

The odor molecules utilized in this work were d-limonene ((*R*)-(+)-limonene, Fujifilm Wako Pure Chemical Co., Ltd., Japan, purity > 95.0%), l-limonene ((*S*)-(-)-limonene, Fujifilm Wako Pure Chemical Co., Ltd., Japan, purity > 98.0%), methyl salicylate (Tokyo Chemical Industry Co., Ltd., Japan, purity > 99.0%), (-)-menthol (l-menthol, (1*R*,2*S*,5*R*)-(-)-menthol, Tokyo Chemical Industry Co., Ltd., Japan, purity > 99.0%), and ethyl propionate (Fujifilm Wako Pure Chemical Co., Ltd., Japan, purity > 97.0%).

### Gas Flow Setup

The homemade gas sensing system using this work is shown in Figure S2. Detailed information about the gas sensing system is provided in the Supporting Information.

### GFET Measurements

GFETs were measured by using a homemade electrical measurement device produced by TOSHIBA Corporation. While drain current was measured, we set the drain voltage (Vd) at 16 or 20 mV, and back gate voltage (Vg) at 0 V. Drain voltage was chosen with the maximum value in the Id measurable range. The real-time response of the drain current was measured every 0.5 s. For data analysis, we used Igor Pro 9 (WaveMetrics) and Python.

### Calculation of gas concentration

Odorant gas concentration was calculated using a saturated vapor pressure. Detailed information is provided in the Supporting Information.

### Calculation of Conductivity Change

We obtained the conductivity change of the GFETs as follows. The average value for one min before the introduction of water vapor or odorant molecule gas was taken as the initial value σ_0_ and the conductivity was normalized by dividing by this value.

### Principal Component Analysis (PCA) and Hierarchical Cluster Analysis (HCA)

PCA and HCA methods were performed using Python (scikit-learn^52^) and Igor 9 (WaveMetrics), respectively. A detailed protocol of PCA and input data are presented in the Supporting Information. For the HCA, the same parameters as in PCA were used (Figure S6). We employed the Euclidean method and the Ward method to calculate the distance between each sample and to form the clusters, respectively.

## Supporting Information Available

Structure of the peptides on the graphite and graphene surface; homemade gas sensing system with graphene field effect transistors (GFETs); chiral recognition of limonene with peptide-functionalized GFETs; long-term observation of limonene sensing; the detection limit of limonene gas using peptide-functionalized GFETs; principal component analysis (PCA) and hierarchical cluster analysis (HCA) (PDF)
