# Supplementary material for: Enantioselective Detection of Gaseous Odorants with Peptide–Graphene Sensors Operating in Humid Environments
Source: ACS Appl Mater Interfaces. 2024 Apr 3;16(15):18564–73. doi: 10.1021/acsami.4c01177 (PMC11040535; doi:10.1021/acsami.4c01177)
Supplement: Supplementary file 1 — am4c01177_si_001.pdf [file am4c01177_si_001.pdf]

Supporting Information for

Enantioselective detection of gaseous odorants with peptide-graphene sensors operating in humid environments

Yui Yamazaki[1], Tatsuru Hitomi[1], Chishu Homma[1], Tharatorn Rungreungthanapol[2], Masayoshi Tanaka[2], Kou Yamada[3], Hiroshi Hamasaki[3], Yoshiaki Sugizaki[3], Atsunobu Isobayashi[3], Hideyuki Tomizawa[3], Mina Okochi[2], Yuhei Hayamizu*,[1]

[1] Department of Materials Science and Engineering, School of Materials and Chemical Technology, Tokyo Institute of Technology, 2-12-1 Ookayama, Meguro-ku, Tokyo, Japan

[2] Department of Chemical Science and Engineering, School of Materials and Chemical Technology, Tokyo Institute of Technology, 2-12-1 Ookayama, Meguro-ku, Tokyo, Japan

[3] Corporate Research & Development Center, Toshiba Corporation,1, Komukai-Toshiba-Cho, Saiwai-ku, Kawasaki 212-8582, Japan

*Corresponding Author:
hayamizu.y.aa@m.titech.ac.jp

Table of contents

Table of Contents

1. Structure of the peptides on the graphite and graphene surface 3

2. Homemade gas-sensing system with graphene field effect transistors (GFETs) 4

3. Chiral recognition of limonene with peptide-functionalized GFETs ... 7

4. Long-term observation of limonene sensing 8

5. The Detection limit of limonene gas using peptide-functionalized GFETs 9

6. Principal component analysis (PCA) and hierarchical cluster analysis (HCA) 11

1. Structure of the peptides on the graphite and graphene surface

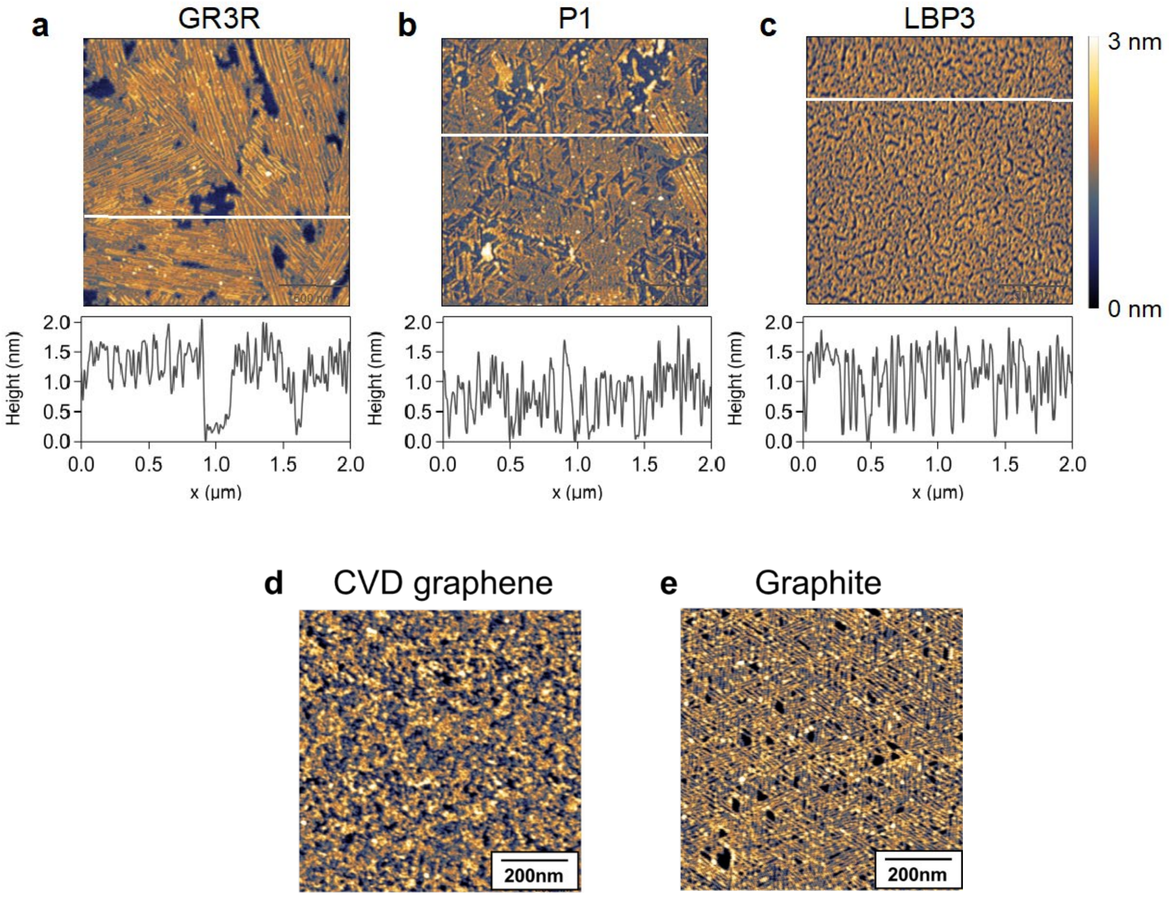

Figure S1. Peptide morphology on the graphite surface. Atomic force microscope (AFM) images of (a) GR3R, (b) P1, and (c) LBP3 peptide at 500 nM. The line profile represents the height of the peptides at the positions of the white lines in the AFM images. The peptide solutions were diluted with DI water and incubated on a graphite surface for one hour. Then, it was dried by N_2 gas blow before the AFM measurements. (d) and (e) are the comparison of the morphology of GR3R peptide on CVD graphene and graphite.

Atomic force microscopy (AFM) images reveal the morphology of self-assembled peptides on surfaces, as shown in Fig. S1. The samples for the AFM measurements were prepared as follows: Aqueous solutions of the respective peptides were prepared at a concentration of 500 nM. These solutions were then placed on either a graphite surface or a CVD graphene surface and incubated for 1 hour in a humid chamber. Subsequently, the solutions were blown off with nitrogen gas to dry the samples, which were then measured by AFM in air.

All peptides, including GR3R, P1, and LBP3, formed thin films with a thickness of around 1.5 nm on graphite surfaces (Fig. S1a-c). Notably, GR3R and P1 exhibited linear patterns in the AFM images (Fig. S1a and b), indicating the formation of ordered peptide structures on the surface. Figures S1d and e provide a comparison between graphene and graphite surfaces. For these images, the peptides were dissolved in a 10-mM phosphate buffer at a concentration of 500 nM. The AFM results revealed that GR3R peptides form thin molecular

films on both graphite and graphene surfaces. However, there was a slight distinction in the linearity of the self-assembled peptide structures; the peptides showed more linear structures on graphite than on graphene. This difference is possibly due to the surface roughness of the SiO_2 layer on the Si wafer, which often causes small undulations in single-layer graphene. Such undulations might affect the formation of long-range ordered structures of peptides on the graphene surface.

2. Homemade gas-sensing system with graphene field effect transistors (GFETs)

Gas flow system

As shown in Figure S2, N_2 gas from one cylinder was divided into three lines of N_2, water vapor, and odor molecules. Mass flow controllers (FCST1005LC-4F2-F10-N2 and FCST1005LC-4F2-F1L-N2, Fujikin, Osaka, Japan) were used to control the gas flow rate. Water vapor was generated by bubbling water with N_2 gas using an impinger (201-060-12-033-27, Savillex, MN, USA). Odor molecules such as D-limonene, L-limonene, methyl salicylate, and ethyl propionate were in liquid form. They were individually absorbed by cotton in an impinger, and their gases were generated by flowing N_2 gas through the cotton. (-)-Menthol gas was produced by placing crystal (-)-menthol in the impinger and flowing N_2 gas. After mixing each gas at an arbitrary flow rate, the mixed gas was introduced into the GFET chip. The GFET chip was placed in a cassette with a gas fluidic channel. The volume of the fluidic channel in the cassette was approximately 24 mm^3. Three cassettes were connected in series to measure three chips simultaneously. The temperature of the measurements was controlled by an incubator at 30°C, and a commercially available humidity sensor (I2C High Accuracy Temp&Humi Sensor SHT35, Seeed Technology Co., Ltd., China) monitored the humidity of the exhaust gas.

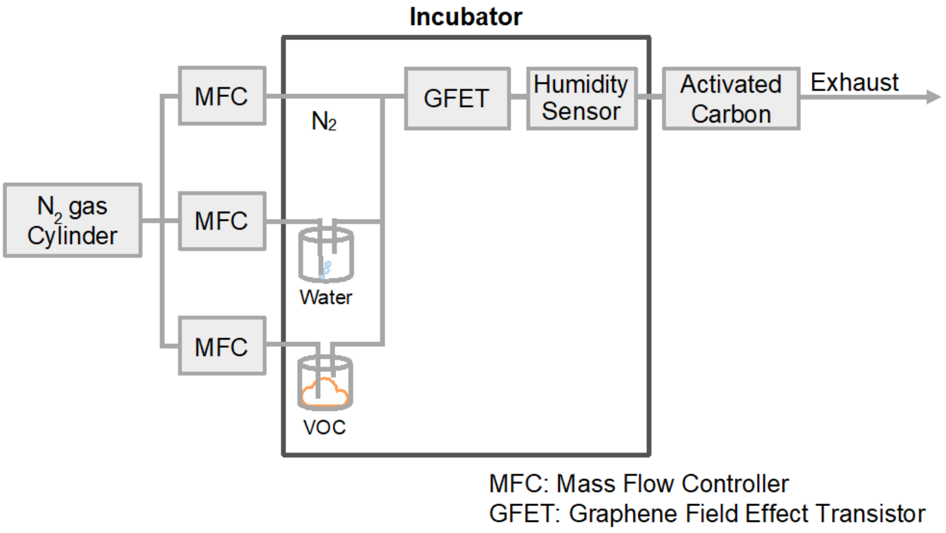

Figure S2. The schematic diagram of the homemade gas sensing system. MFC represents mass flow controllers.

Gate response of GFET device

We investigated the gate response of the untreated GFET under N_2 gas. Before starting the gate response measurement, 100 sccm of N_2 gas was flown to dry the graphene surface. The gate voltage was applied from the silicon wafer through the SiO_2 layer, and the source-drain current was measured. Figure S3 black line shows the gate response. The charge neutral point of this GFET is located at more than 50 V. In the gas sensing measurements, the gate voltage was zero. After measuring the gate response of untreated graphene, 500 nM GR3R peptide was incubated for 1 hour, and the peptide solution was blown dry with N_2 gas. It was set in the instrument again and the gate response was measured. The result was the orange line in Figure S3.

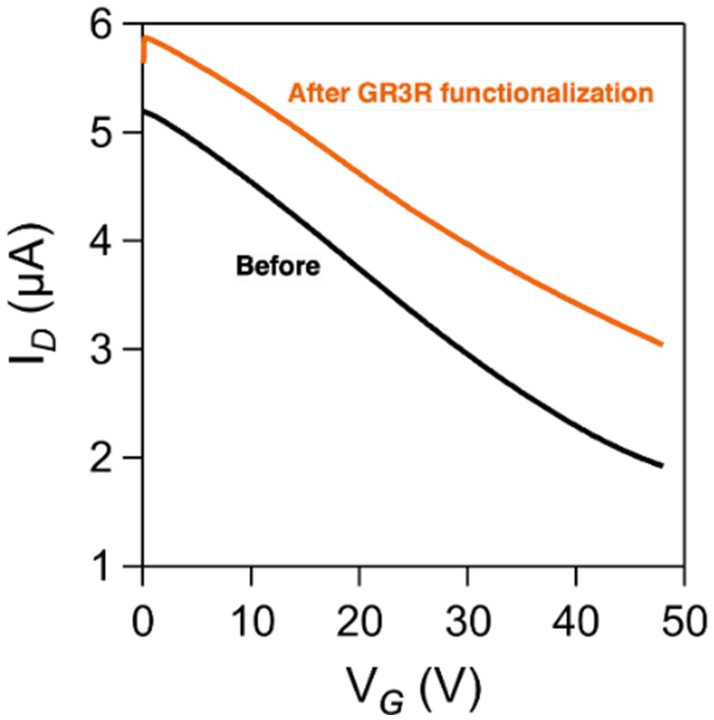

Figure S3. Averaged gate response of before and after 500nM-GR3R functionalized GFET under N_2 conditions. The line represents the mean value of the data point.

Peptide morphology on the graphite surface under different humidity conditions

The effects of water vapor on self-assembled peptides were investigated through environmental measurements using atomic force microscopy (AFM). The experiments were conducted using an AFM equipped with a gas chamber (Agilent AFM 5500). This setup featured a nitrogen gas source connected to a water-bubbling system, enabling the generation of N_2 gas at controlled humidity levels. GR3R peptides were initially self-assembled on a graphite surface and then dried using nitrogen gas. The surface was characterized by AFM prior to increasing the humidity up to 60% by introducing N_2 gas infused with water vapor. As shown in Fig. S4a, the AFM images reveal self-assembled peptides predominantly in the central region of interest. A relatively low concentration of peptide solution was used to limit the surface coverage of the self-assembled peptides, allowing for precise measurement of the height of the self-assembled structure from the bare graphite surface. Fig. S4b presents the line profile of these peptides, indicating that the thickness of the peptide layer increased by 10 to 20% when the environmental humidity was raised to 60% relative humidity (Fig. S4). This thickness variation suggests that the peptide layer is capable of absorbing water molecules to a certain extent while maintaining its unique structural properties. As a result, the layer can sustain a relatively stable amount of absorbed water under standard environmental humidity conditions. This characteristic is crucial for ensuring the stability of conductivity in varying humidity environments.

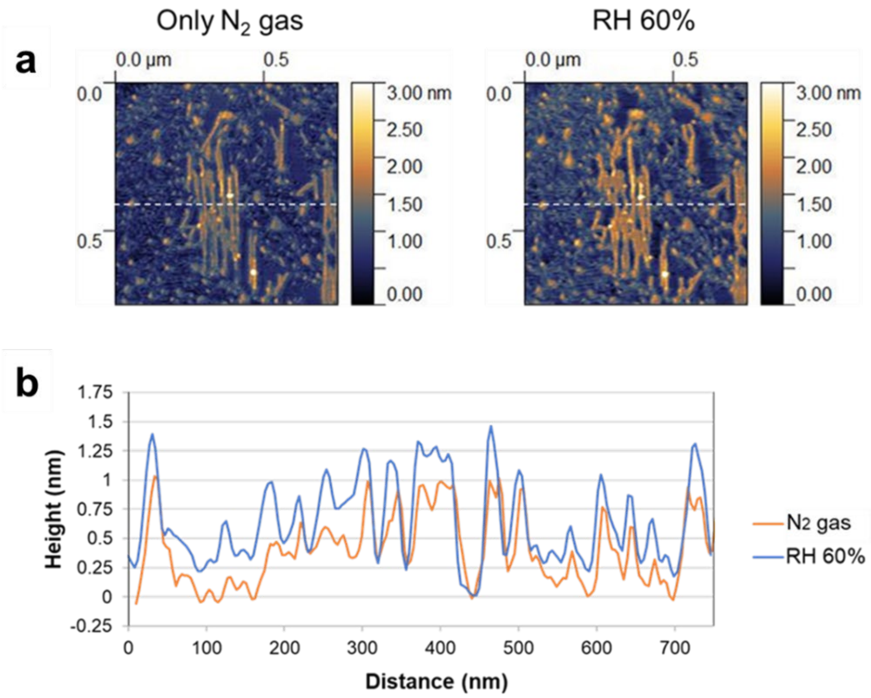

Figure S4. Atomic force microscopy (AFM) images showing the peptide morphology (a) and its height profile (b) on the graphite surface under pure N_2 and 60% relative humidity (RH) conditions.

3. Chiral recognition of limonene with peptide-functionalized GFETs

Figure S4 illustrates the real-time current response of peptide-functionalized GFETs in detecting both D- and L-limonene under conditions of 53% RH and N_2 gas. The y-axes represent the absolute current values for each measurement. Figures S5a and S5b display results obtained under humid conditions. The electrical signal from the untreated GFET (Fig. S5a) was more pronounced than that from GFETs functionalized with LBP3 peptides (Fig. S5b). This heightened response is attributed to the direct adsorption of target molecules onto the graphene surface, significantly influencing its conductivity. In contrast, GFETs functionalized with LBP3 peptides exhibited a less marked change in conductivity. The peptides serve as an intermediate layer, moderating the interaction between the target molecules and the graphene surface, resulting in a smaller conductivity change in peptide-functionalized graphene compared to untreated GFETs. Although the absolute signal of the peptide-functionalized GFETs is slightly lower than that of the untreated GFETs, the LBP3 peptides achieve high selectivity against D-limonene.

In tests conducted under N_2 gas without water vapor (Fig. S5c and S5d), there was a drastic change in the absolute signal value compared with the ones under humid conditions. The untreated GFETs displayed weaker signals than those of LBP3-functionalized GFETs. This suggests that the dipoles of water molecules adsorbed on the graphene surface play a significant role in generating polarization at the graphene interface. Furthermore, the LBP3 peptides facilitate selective detection against D-limonene.

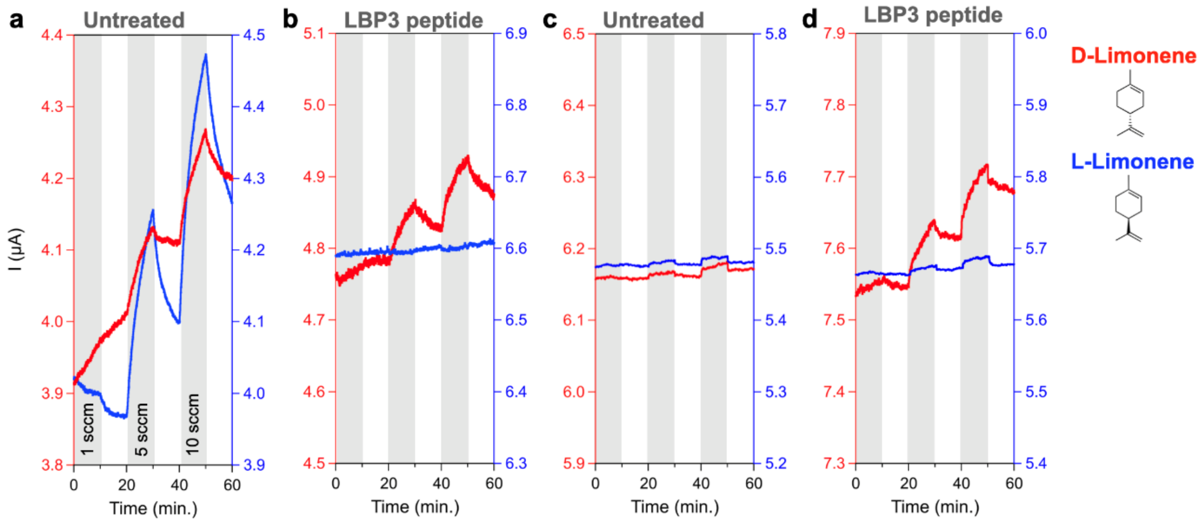

Figure S5. Chiral recognition by GFETs functionalized with peptides. Current change of GFETs responding to enantiomers of limonene under 53% RH condition and N_2 gas atmosphere. The curves show the responses of GFETs to D-limonene (red) and L-limonene (blue). It highlights the differences in chiral selectivity under 53% RH conditions between (a) untreated GFETs and (b) GFETs functionalized with LBP3 peptides. Panels (c) and (d) illustrate the current responses under an N_2 gas atmosphere for each GFET type, respectively. These results represent the original data for Fig. 3, prior to the normalization of conductivity.

4. Long-term observation of limonene sensing

To deepen our understanding of the response of LBP3 peptide-functionalized GFETs to D-limonene, we conducted an extended study over a longer timeframe. Similar to prior experiments, we exposed the GFET to carrier N_2 gas at 53% RH to establish a stable conductivity baseline. Once a steady state was achieved, a 10 sccm flow of D-limonene gas was introduced for 100 minutes, creating the "ON" state. This was followed by a 150-minute purging process using carrier N_2 gas at 53% RH, constituting the "OFF" state.

As depicted in Fig. S6, a rapid increase in conductivity was observed within the first 15 minutes after introducing D-limonene gas. This initial surge was followed by a temporary deceleration in the rate of conductivity change until around 40 minutes. Beyond this point, the conductivity began to increase again, with its slope becoming nearly constant. The changes observed up to 40 minutes are likely attributable to the binding of individual limonene molecules to the peptide probes. The continued increase in conductivity post-40 minutes could result from the ongoing accumulation of hydrophobic limonene molecules, potentially leading to their aggregation. During the "OFF" state, the conductivity did not return to zero within 150 minutes, suggesting a strong interaction between the LBP3 peptides and D-limonene molecules that contributed to the persistent conductivity changes. The selection of LBP3 peptide as a strong binder to D-limonene appears to be justified based on these results.

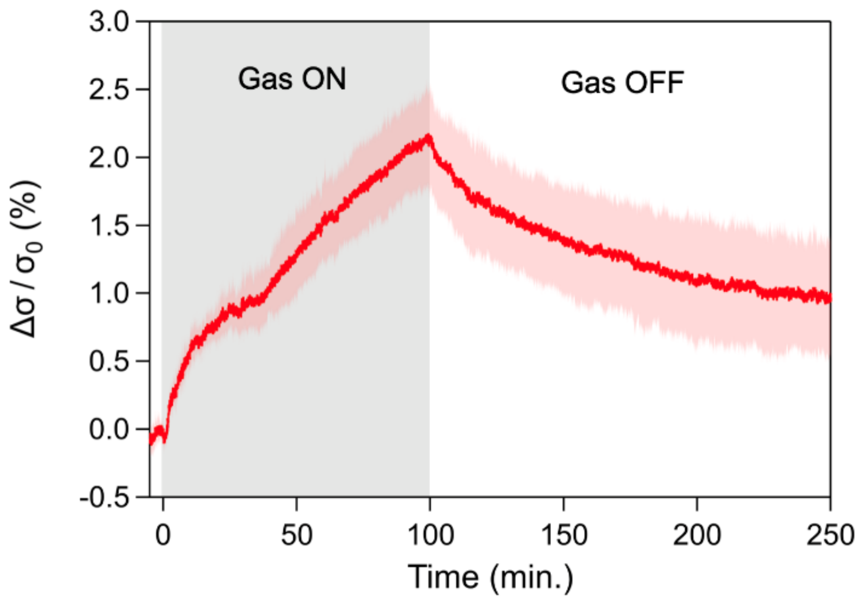

Figure S6. Response to long-term exposure of D-limonene to GFET-functionalized with LBP3 peptides under 53% RH conditions. The solid curve and colored shadow represent the mean value of the conductivity change and its standard deviation, respectively.

5. The Detection limit of limonene gas using peptide-functionalized GFETs

Calculation of D-limonene gas concentration

The concentration of the odorant gas was estimated from the saturated vapor pressure P_{VOC} of each odorant molecule by following previous work[1]. P_{VOC} was obtained from PubChem (https://pubchem.ncbi.nlm.nih.gov/). The saturated vapor concentration (ppm) was calculated using the following equation.

$$\text{Saturated vapor concentration (ppm)} = \frac{P_{\text{VOC}}\ \text{(Pa or mmHg)}}{P_{\text{Air}}\ \text{(Pa or mmHg)}} \times 10^6 \quad (1)$$

P_{Air} is the pressure of the air. The VOC gas concentrations injected into GFETs were calculated using equation (2).

$$\text{Estimated VOC gas concentration (ppm)} = \frac{\text{Flow rate}_{\text{VOC}}}{\text{Total flow rate}} \times \text{Saturated vapor concentration (ppm)} \quad (2)$$

The Flow rate $_{VOC}$ represents the flow rate of the odorant gas. The total flow rate is the sum of carrier gas and odorant gas. The estimated concentrations of each odorant at the flow rate of 10 sccm are summarized in Table S1.

Table S1. The concentration of each odorant at the flow rate of 10 sccm.

Odorants	Concentrations at 10 sccm (ppm)
D-Limonene	170.47
L-Limonene	172.25
(-)-Menthol	9.57
Methyl salicylate	5.38
Ethyl propionate	4282.30

The detection limit of GFETs functionalized with peptides

To estimate the detection limit, we first calculated the noise level. The data from five channels were used to calculate the noise level of the GFET sensor. The noise level was defined by the root mean square of data points of the baseline. The noise level was 0.06% in the conductivity change. The limit of detection (LOD) of the GFET sensor was calculated by using equation (3).

$$\mathrm{LOD} = \frac{\text{Noise level}}{\text{Slope}} \quad (3)$$

The response of each GFET to D-limonene under the conditions of N_2 and RH 53% is plotted in Figure S5. The dashed lines provide the slope used in equation (3). Table S2 summarizes the extracted LOD against D-limonene gas for each device.

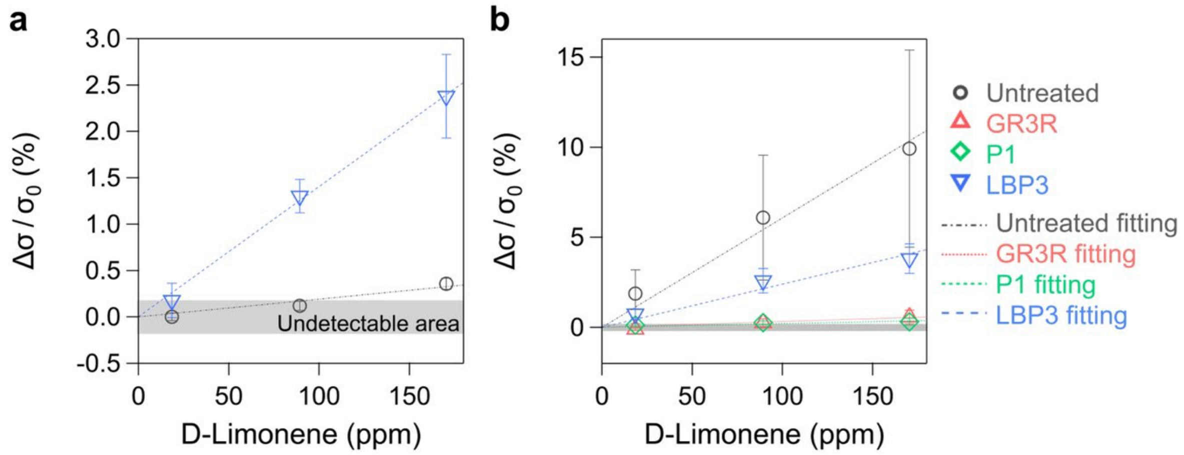

Figure S7. Estimation of the limit of detection. The plot of the observed magnitudes of conductivity change versus D-limonene gas concentration under (a) N_2 and (b) 53% RH conditions. The data are taken from Figure 4a-d and Figure S4. The markers represent the mean value. Error bars indicate the standard deviation of the data point. The dashed lines are fitted with a linear function.

Table S2. Limit of detection (LOD) of peptide-functionalized GFETs response to D-limonene gas.

Surface Functionalization of GFETs	Limit of Detection (LOD)	
	Under N_2 (ppm)	Under 53% RH (ppm)
Untreated	105.4	3.0
GR3R	-	56.6
P1	-	85.8
LBP3	12.8	7.5

6. Principal component analysis (PCA) and hierarchical cluster analysis (HCA)

The eight data points were used as input data for PCA and HCA, as shown in Figure S6. Data points 1, 3, 5, and 7 are the slopes of the conductivity changes over time. Data points 2, 4, 6, and 8 are the magnitudes of the conductivity changes.

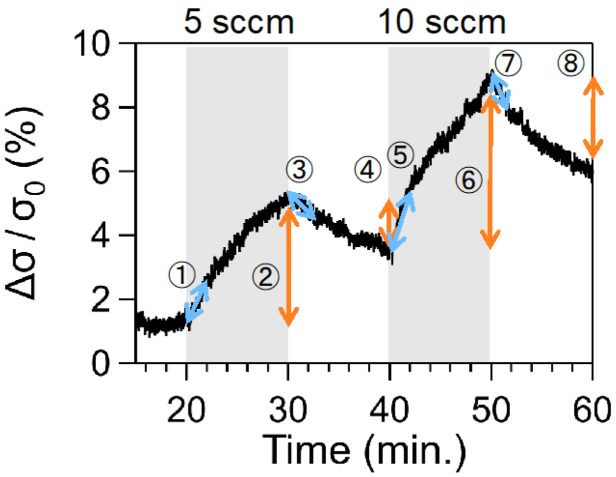

Figure S8. Schematic representation of the extracted feature values from the GFET response.

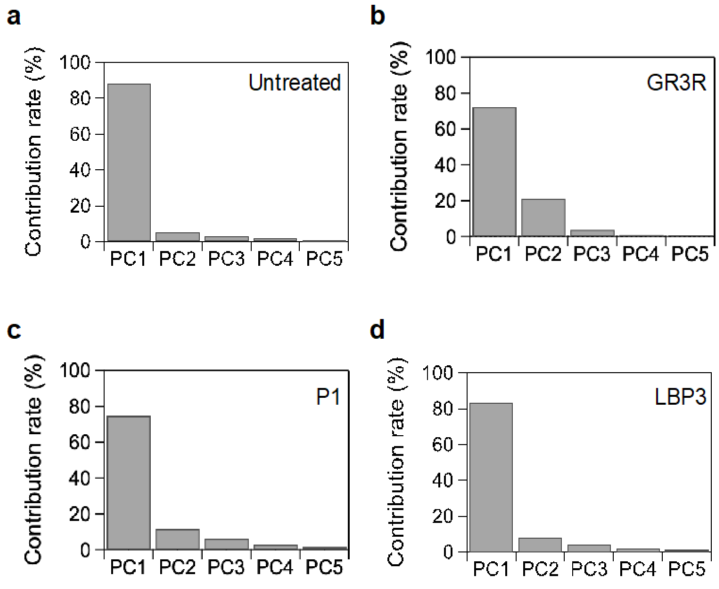

Figure S9 Contribution ratio of PC1-PC5 components with each surface modification of GFETs. Each plot represents (a) untreated, (b) GR3R, (c) P1, and (d) LBP3, respectively.

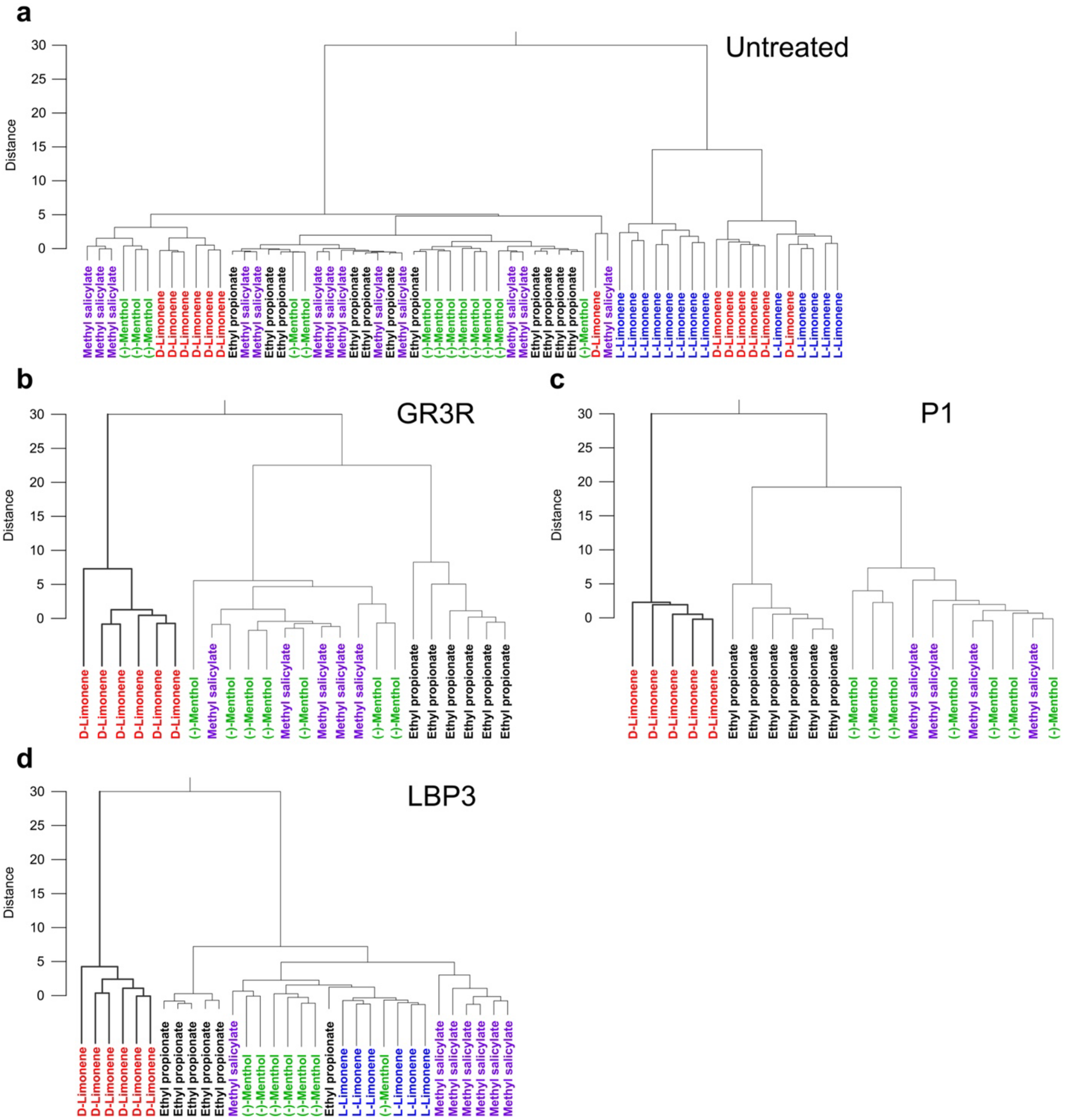

Figure S10. Dendrogram of each surface condition generated by hierarchical cluster analysis (HCA). Each plot represents (a) untreated, (b) GR3R, (c) P1, and (d) LBP3, respectively.

References

(1) Hassan, K.; Tung, T. T.; Yap, P. L.; Rastin, H.; Stanley, N.; Nine, M. J.; Losic, D. Fractal Design for Advancing the Performance of Chemoresistive Sensors. *ACS Sens* **2021**, *6* (10), 3685–3695.
